# Assessing Uncertainty in A2 Respiratory Syncytial Virus Viral Dynamics 

**DOI:** 10.1155/2015/567589

**Published:** 2015-09-14

**Authors:** Gilberto González-Parra, Hana M. Dobrovolny

**Affiliations:** ^1^Department of Physics and Astronomy, Texas Christian University, Fort Worth, TX 76132, USA; ^2^Grupo de Matemática Multidisciplinar (GMM), Universidad de Los Andes, Mérida 5101, Venezuela

## Abstract

Respiratory syncytial virus (RSV) is the most common cause of bronchiolitis and pneumonia in children younger than 1 year of age in the United States. Moreover, RSV is being recognized more often as a significant cause of respiratory illness in older adults. Although RSV has been studied both clinically and in vitro, a quantitative understanding of the infection dynamics is still lacking. In this paper, we study the effect of uncertainty in the main parameters of a viral kinetics model of RSV. We first characterize the RSV replication cycle and extract parameter values by fitting the mathematical model to in vivo data from eight human subjects. We then use Monte Carlo numerical simulations to determine how uncertainty in the parameter values will affect model predictions. We find that uncertainty in the infection rate, eclipse phase duration, and infectious lifespan most affect the predicted dynamics of RSV. This study provides the first estimate of in vivo RSV infection parameters, helping to quantify RSV dynamics. Our assessment of the effect of uncertainty will help guide future experimental design to obtain more precise parameter values.

## 1. Introduction

Respiratory syncytial virus (RSV) is a major cause of lower respiratory tract disease among infants, a frequent pathogen in elderly and immunosuppressed patients, and a major public health concern worldwide [1–5]. Healthy adults who contract RSV experience mild or asymptomatic infections, but it is the cause of 18% of hospitalizations due to pneumonia in adults older than 65 [6]. There is currently no vaccine for RSV and drug treatment is largely limited to treatment of symptoms and supportive care [7]. Thus it is crucial that we develop a detailed understanding of the viral kinetics of this disease.

Historically, mathematical and computational methods have not played a large role in immunology and virology. This is now changing, and impressive advances have come from the use of simple models applied to the interpretation of quantitative data [8]. Mathematical models of viral infections help us quantify key parameters of the infection process [9–12], optimize drug treatment regimens [13–16], and understand complex host-virus interactions [17–19]. Mathematical modeling is now commonly used to study HIV [20] and is becoming more common in other serious viral infections such as influenza [21] and HCV [22]; it has not yet been used to investigate respiratory syncytial virus (RSV).

Initial modeling studies for any virus often use a system of nonlinear differential equations and the models are typically fit to viral time course data to generate estimates of viral kinetic parameters. However, given the large amount of experimental error in viral titer measurements [23], parameter estimates vary widely and contain some degree of uncertainty. Uncertainty in model parameters can limit the predictive ability of the model, so it is important to understand how parameter uncertainty alters the predicted time course of the model.

Uncertainty in differential equations has been considered in recent decades in a wide variety of applied areas, such as physics, chemistry, biology, economics, sociology, and medicine [24–26]. Uncertainty in a differential equation model can arise either through uncertainty in the initial conditions or through uncertainty in equation coefficients. In this paper uncertainty of both types is considered in the context of a viral kinetic model. Studies of the impact of parameter distributions on epidemic models [26] have proved to be useful in determining upper and lower estimates of the size of an epidemic.

This paper is organized as follows.  Section 2 presents the viral kinetic model, fitting procedure, and Monte Carlo method. In Section 3, we present the fits of the viral model to data from eight patients infected with the A2 strain of RSV. Additionally, results of the estimated parameters and the Monte Carlo simulations are presented. Discussion and conclusions are presented in Section 4.

## 2. Methods

### 2.1. Patient Data

The experimental data used in this paper was first published in Lee et al. [27]. Briefly, 12 healthy young adults were challenged with the A2 strain of RSV. Nasal washes were performed daily for twelve days after inoculation. Nasal washes were cultured in HEp-2 cells to determine viral titer in each nasal wash sample. Of the twelve patients challenged with RSV, only eight showed elevated viral load for several days and were suitable for fitting and parameter extraction.

### 2.2. Model

In this paper we will use a model based on an autonomous system of nonlinear ordinary differential equations to characterize the in vivo infection dynamics of the A2 strain of RSV. The model is an extension of the basic viral infection model for influenza described in [9]

(1) dTdt=−βNTVdE1dt=βNTV−nEτEE1dEjdt=nEτEEj−1−nEτEEjfor  j=2,…,nEdI1dt=nEτEEnE−nIτII1dIjdt=nIτIIj−1−nIτIIjfor  j=2,…,nIdVdt=p∑j=1nIIj−cV.

**Figure 1:**
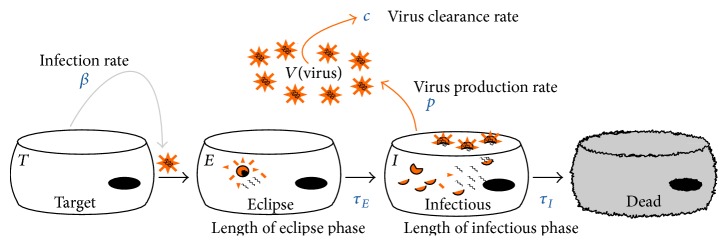
Viral kinetic model. The virus, *V*, attacks target cells, *T*, at rate *β*. Once infected, target cells enter the eclipse phase, *E*. The eclipse phase lasts an average time of *τ*_*E*_, after which the cells become infectious cells, *I*. The infectious cells produce new virions at rate *p*, and the virus decays at rate *c*. The cells remain infectious for an average time of *τ*_*I*_, after which they become dead cells.

In this model, uninfected target cells, *T*, are infected at a rate *β* when they encounter a virion *V*. The cells transition to the eclipse phase, *E*, where they are infected but not yet actively producing virions. After an average time *τ*_*E*_, the cells transition to the infectious phase, *I*, where they actively produce virus at a rate *p*. After an average time *τ*_*I*_, infectious cells die. Virus is cleared from the system at a rate *c*. The transitions between eclipse/infectious cells and infectious/dead cells are modelled as gamma distributions, as done by Pinilla et al. [12], since exponential and hard delays are known to be biologically unrealistic [28–30]. *n*_*E*_ and *n*_*I*_ are the number of compartments used to represent the eclipse phase and infectious phase, respectively. A schematic of the model is shown in Figure 1. This model can give us insight into the infection process since we can extract parameters such as the duration of the eclipse phase and the lifespan of an infected cell. Unfortunately, the model has seven parameters and so requires a rather large and complete data set.

### 2.3. Fitting Algorithms

In order to characterize the RSV replication cycle and extract kinetic parameter values, we need to fit the mathematical model to the patient data [27]. We determine the best fit by minimizing the sum of squared residuals (SSR). The initial parameter space is large due to the seven unknown parameters of model (1). Moreover, since the initial viral load is unknown this adds an extra parameter to the search space. In this way, to obtain realistic parameter values, we transform the original fit problem into a constrained nonlinear optimization problem by restricting the parameter search space to biologically realistic values. While the parameter space was restricted, the boundaries are large. The range of values used for each parameter is as follows. For the virus production rate *p* and the infection rate *β*, we assume a wide range since the units of both parameters include units of viral titer, which are not standardized and are known to vary from one experiment to another [31]. The range for *p* in the restricted fitting process is [10^−10^, 10^16^]  TCID_50_/mL/(cell · d). For the infectious rate *β*, the range is [10^−10^, 1]  (TCID_50_/mL)^−1^ · d^−1^. The range for the viral clearance rate was restricted to [10^−4^, 10^2^]/d. The range for the mean infectious cell lifespan, *τ*_*I*_, was limited to [0.001,1]d. The same range was assumed for the mean duration of the eclipse phase *τ*_*E*_. For the parameters *n*_*E*_ and *n*_*I*_ the literature is very scarce as these parameters are more related to the shape of the probability distribution of the infectious and eclipse phases, respectively. It is important to remark that these parameters do not change the mean duration of phases, just the variance. In our particular case we allowed a range of [1,100]. Finally, for the initial viral load, again we used a not too restrictive range in order to have large search parameter space, [10^−2^, 10^16^]  TCID_50_/mL. We used the Nelder-Mead algorithm to minimize the SSR [32] within the restricted parameter space.

### 2.4. Viral Kinetic Parameters

It is sometimes difficult to compare parameter estimates from different experiments since the units of viral titer depend on the details of measurement. There is no universal standard viral titer unit, making comparison of parameters such as *p* and *β* irrelevant. Instead, we will focus on parameters which have a universal standardized unit (units of time, in this case). In addition to the mean duration of the eclipse phase *τ*_*E*_ and the mean duration of the infectious phase *τ*_*I*_, we can compare the following parameters:

(i) $t_{inf}=\sqrt{2/p\beta }$: the infecting time is the mean time for an infected cell to infect a neighbouring cell.
(ii) $\sigma_{E}=\tau_{E}/\sqrt{n_{E}}$: this is the standard deviation in the duration of the eclipse phase.
(iii) $\sigma_{I}=\tau_{I}/\sqrt{n_{I}}$: this is the standard deviation in the duration of the infectious phase.

### 2.5. Monte Carlo Assessment of Uncertainty

The reliability of the parameter estimates depends mainly on two factors. The first one is the accuracy of the experimental data, which is out of our hands, but it is known that these types of viral data are subject to uncertainty [23]. Inaccurate or wildly varying data will affect both the estimated parameter values and the reliability of the viral dynamics prediction. The second factor is the ability of the fitting method to find the true best fit values. While we cannot judge how much each of these factors affects the resulting parameter estimates, it is clear that there will be some error in the estimates. One of the aims of this paper is to examine how changes in the estimated values of the model parameters will affect predicted viral dynamics of RSV.

We have chosen to study the effect of parameter uncertainty on viral dynamics using a Monte Carlo method. The Monte Carlo method allows us to study random effects with different distributions in ordinary differential equation models. We employ a conceptually simple Monte Carlo approach by running numerical simulations separately for each parameter which results in a set of corresponding plausible simulation predictions. These predictions can be used to characterize the uncertainty in viral titer time course due to inaccuracy in either single parameters or combinations of parameters [26, 29].

## 3. Results

### 3.1. Parameter Estimates for RSV Infected Patients

**Figure 2:**
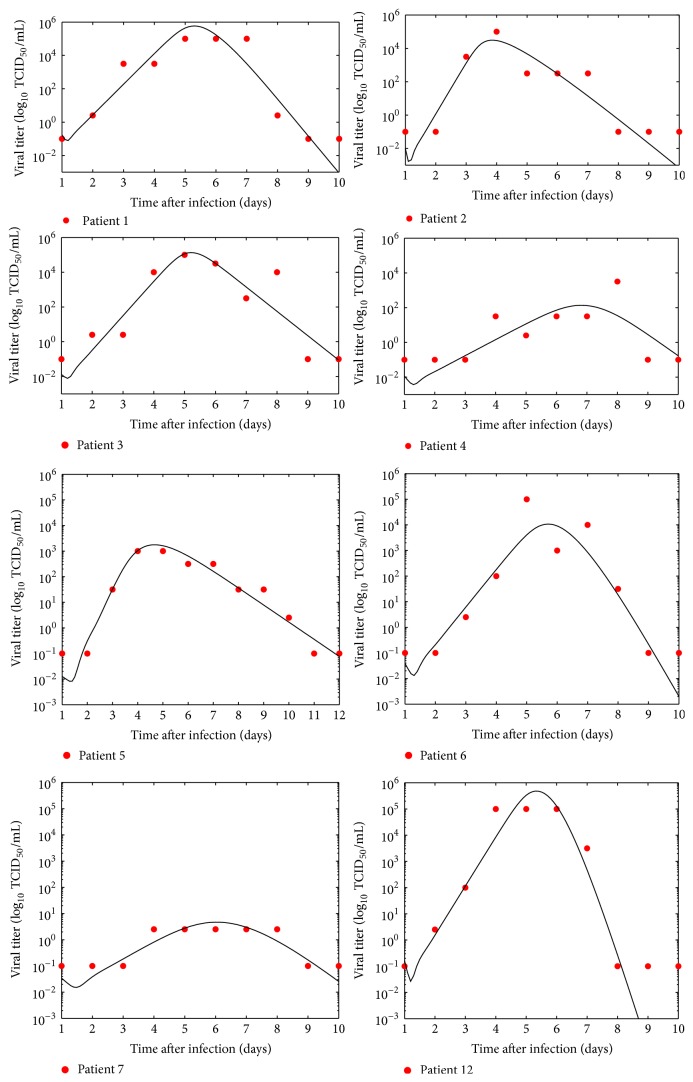
Numerical simulations of the virus kinetic model (1) fitted for different patients. The graphs present viral titers in TCID50/mL of nasal wash (red circles) and the fits.

One of our aims is to estimate the parameters of the mathematical model (1) by fitting it to RSV patient data.  Figure 2 presents patient viral titers and the best fits of (1) for the different patients with corresponding viral kinetic parameter estimates presented in Table 1. We can see that the model does an adequate job of fitting patient data, but given the large number of free parameters and the limited size of each data set, this is not particularly surprising.

Despite the overfitting, the extracted parameter values seem to be biologically reasonable. While we have no previous RSV data for comparison, we can examine our parameter estimates in the context of what has been found for influenza. Influenza is also an upper respiratory tract viral infection that typically causes mild disease in healthy adults, so the two infections have been compared before [33]. In vivo challenge studies indicate that influenza viral loads peak at about 2 dpi with symptoms peaking about a day later. RSV viral load peaks at about 5-6 dpi with symptoms peaking around 6 dpi. Given these results, we would generally expect RSV infection processes to take longer than similar processes in influenza.

Several studies have determined estimates of the infecting time for influenza ranging from 0.02 h to 2.5 h [9, 12, 19, 34–36]. Our estimates have values in the range of 0.72 h to 6.2 h which overlap with some estimates of influenza infecting time but also tend to be somewhat longer. Estimates of the duration of the eclipse phase *τ*_*E*_ for influenza range from 1 h to 9 h [9, 12, 19, 30, 34, 37–39]. Our estimates for the duration of the eclipse phase of RSV range from 7.2 h to 24 h. With one exception, these estimates are longer than the estimated eclipse durations for influenza. Finally, the infectious lifespan of influenza-infected cells is estimated to range between 6 h and 49 h [9, 12, 30, 39, 40]. Our estimate for the infectious lifespan of RSV infected cells is 4.8 h to 18 h. The RSV lifespans are on the shorter end of the estimates for influenza.

One parameter for which we have some RSV data for comparison is the viral clearance rate. Viral decay rate can be determined from mock infection experiments. In these experiments, the virus is placed in a dish without cells and infectious virus is measured every few hours. Mock infection experiments for RSV indicate clearance rates in the range of 0.016–0.034 /h [41–43]. Our estimates in the range 0.063–0.73 /h are higher than those determined from mock infection experiments. This is likely due to the effect of the immune response which helps clear virus in the human body effectively increasing the in vivo clearance rate.

Finally, we determined estimates for the standard deviation in the eclipse duration and the infectious lifespan. There are not many estimates of these parameters, even for influenza, since most models assume exponential transitions between the phases of the cell life cycle. Our estimates of the standard deviation in infectious lifespan of RSV infected cells range from 2.2 h to 10 h and our estimates of the standard deviation of the eclipse duration during RSV infection range from 4.8 h to 11 h. For influenza, infectious lifespan standard deviation estimates range between 1.4 h and 9.7 h [12, 30] and eclipse duration standard deviation estimates range from 0.15 h to 4.6 h [12, 30]. Standard deviation in the duration of the eclipse phase of RSV infections is typically larger than the standard deviation in eclipse phase of influenza infections. Estimates of the standard deviation in infectious lifespans of both RSV and influenza largely overlap.

### 3.2. Monte Carlo Simulations for Sensitivity Analysis of the Parameters

**Figure 3:**
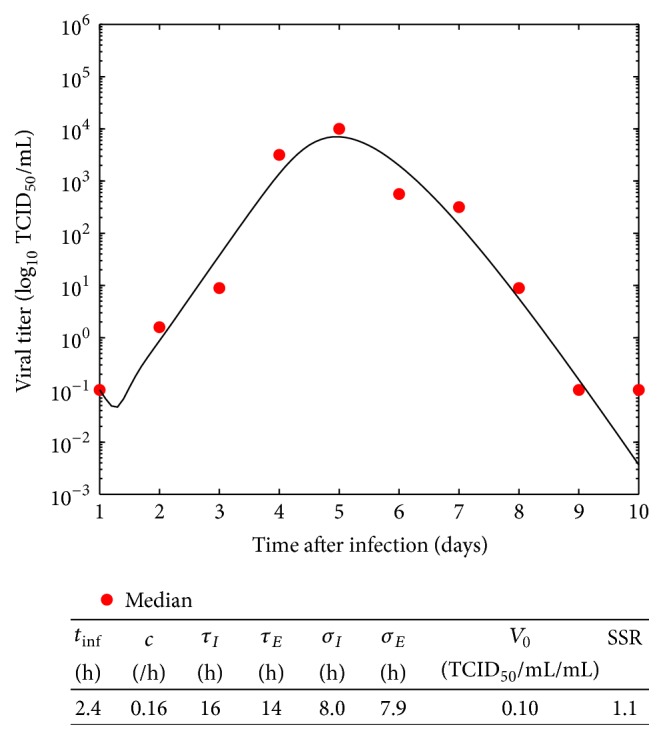
Fit of the viral kinetics model (1) to median RSV in vivo data.

Given the range of estimated parameter values and the known experimental error in viral titer measurements [23], we need to understand how uncertainty in our parameter estimates translates into uncertainty in the viral titer time course. As a demonstrative example, we use a fit of the viral kinetic model to median values of the viral titers from the eight patients. The data, model fit, and parameter estimates are shown in Figure 3.

**Figure 4:**
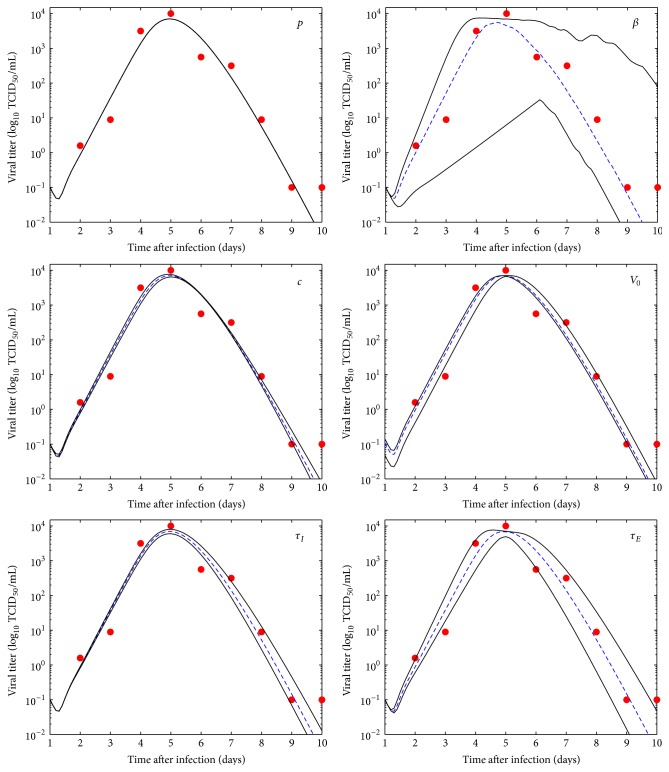
Monte Carlo numerical simulations of the virus kinetic model using the parameter values extracted from the fit to the median virus data. The graphs present the mean (dashed blue line) and 95% confidence interval (solid black lines).

To begin the Monte Carlo process, we need to assume probability density functions for each of the parameters. In this case, we assumed that the parameters follow a Gaussian distribution. The mean of the Gaussian distribution is set to be the parameter value determined through fitting of the median data set. The variance is assumed to be proportional to the mean value of the distribution, *σ* ^2^ = *kμ*, where *k* = 0.01 (with the exception of *β* where this resulted in excessively large confidence intervals, so *k* was set to 0.001). In order to avoid negative values, we have allowed for truncated Gaussian distributions for some parameter values. Monte Carlo simulations are performed independently for each of the following parameters: production rate *p*, infection rate *β*, viral clearance rate *c*, initial viral load *V*_0_, mean eclipse duration *τ*_*E*_, and mean infectious lifespan *τ*_*I*_. Monte Carlo numerical simulations are carried out with *n* = 100 samples for each parameter to obtain a series of viral titer curves. The 95% confidence intervals are determined and shown in Figure 4 as solid black lines. We must be careful when interpreting these lines: a 95% confidence interval does not mean that for a given realized interval calculated from sample data there is a 95% probability the population parameter lies within the interval or that there is a 95% probability that the interval covers the population parameter. The 95% probability relates to the reliability of the estimation procedure; that is, 95% of the generated intervals would contain the true value [44].

Figure 4 clearly shows that our model predictions are extremely sensitive to changes in *β*, the infection rate. Not only is the model highly sensitive to changes in *β*, but changes in *β* lead to uncertainty throughout the entire viral titer curve. This is not entirely surprising since *β* characterizes the first step in the infection cycle, so any deviation in the early stages of the infection process will be transmitted through later stages. The only other variable that exhibits uncertainty larger than the typical experimental error of viral titer measurements is *τ*_*E*_, the mean eclipse duration. In this case, uncertainty in *τ*_*E*_ leads to small changes in the growth phase of the infection and results in larger changes in the decay phase. Uncertainty in the remaining parameters leads to changes in model predictions that are undetectable given the error in viral titer measurements. It is interesting to note, however, how uncertainty in particular parameter values manifests itself in the viral titer curve. Uncertainty in *V*_0_ leads to a temporal shift in the viral titer curve. Uncertainty in *τ*_*I*_ and *c* leads to changes primarily in the decay phase of the infection. Somewhat surprisingly, our model seems to be very insensitive to changes in the production rate, *p*, showing no changes in the resulting curve as *p* is varied.

## 4. Discussion and Conclusions

This paper presented the first fits of a viral kinetics model to in vivo RSV infections. This allowed us to extract viral kinetic parameters for an in vivo RSV infection. While it is difficult to judge the accuracy of our parameter estimates since there are no similar studies for RSV, we can compare RSV parameter estimates to parameter estimates for influenza. Both diseases are viral infections of the respiratory tract that most often cause mild illness but have the potential to cause serious illness and death. A previous study comparing the two infections in an in vivo challenge study noted that influenza viral load peaked about 3 dpi before RSV and that RSV appeared to have a longer incubation period. These findings agree with our quantitative findings of a longer infecting time and longer eclipse phase duration for RSV than for influenza. Our study also suggests, however, that the infectious cell lifespan is shorter for RSV than for influenza, showing that not all processes take longer for RSV. Some of this decreased lifespan in RSV could potentially be accounted for by the actions of cytotoxic T lymphocytes (CTLs). CTLs kill infected cells, but it takes several days after infection (~5–8 dpi) for them to appear in substantial numbers [17, 45]. With the longer incubation period of RSV, driven by both the longer infecting time and the longer eclipse duration, there will be more CTLs available to kill cells when they finally do become infected, effectively lowering the mean infectious lifetime. Another possible reason for the shorter infectious lifespan is the formation of syncytia during RSV [46]. When already infectious cells fuse with uninfected cells, it is not known whether the newly formed syncytium will have a different effective lifespan. The effect of syncytia on the viral time course is, as yet, unclear and should be investigated further.

This paper also investigated the effect of parameter uncertainty on RSV dynamics. It is important to understand the limits of the predictive capabilities of mathematical models since they are more often being used to assess the effect of vaccines and drug treatment regimens [47]. Our Monte Carlo simulation method allows us to investigate the effect uncertainty in each parameter has on the viral time course. We found that the model was most sensitive to changes in the infection rate *β* and that changes in *β* are transmitted through the entire viral time course. This suggests that we must be particularly careful in trying to extract values for *β*. It has been shown that including both infectious viral titer and RNA measurements will reduce the uncertainty in estimated parameters for influenza [48] and a similar approach would certainly work for RSV. Alternatively, experiments could be designed to directly measure the infection rate [49], likely reducing uncertainty in the measurement.

While we have used individual fits to patient data to estimate RSV parameters, other methods may be used to estimate the parameter values. Mixed effect models are increasingly used to estimate parameters [50] for infectious disease models, particularly in cases where data might be scarce or incomplete. These models have several underlying assumptions and they are useful when repeated measurements are made on the same statistical units. It is particularly difficult to use the mixed effect framework on measurements of viral titer since the viral measurement unit is not standardized, so we cannot assume that similar measurements from different patients actually represent the same amount of virus. Also, to the best of our knowledge applying that method to assess the effect of each parameter on the dynamics of the variables is not straightforward. On the other hand, Monte Carlo method has been used widely to assess the effect of parameter's uncertainty.

While this paper focused on fitting a particular data set for RSV, the assessment tools presented here will be widely applicable to other systems of differential equations. The Monte Carlo assessment of the effect of parameter uncertainty can guide experimentalists in developing experiments that will produce more accurate predictive models.

## Figures and Tables

**Table 1:** Estimated parameters for RSV in vivo infection.

Patient^*∗*^	*t*_inf_	*c*	*τ*_*I*_	*τ*_*E*_	*σ*_*I*_	*σ*_*E*_	*V*_0_	SSR
	(h)	(/h)	(h)	(h)	(h)	(h)	(TCID_50_/mL/mL)	
1	2.4	0.28	7.2	7.2	5.1	5.1	0.17	5.6
2	4.3	0.78	5.3	19	2.2	11	0.010	5.3
3	1.9	0.13	4.8	9.6	2.8	4.8	0.013	9.5
4	3.8	0.20	7.9	11	5.6	7.8	0.011	8.8
5	0.72	0.063	18	24	10	9.1	0.010	1.1
6	2.4	0.20	10	17	4.5	8.5	0.012	4.3
7	6.2	0.096	14	17	9.9	9.8	0.030	0.6
12	1.7	0.44	13	10	3.4	5.0	0.16	2.3

Median	2.4	0.20	9.0	14	4.8	8.2	0.13	4.8

^*∗*^Patient numbers are those originally found in [27].
